# Utilization of pre-hospital pelvic circumferential compression devices for pelvic fractures: survey of U.S. level I trauma centers

**DOI:** 10.1186/s13037-020-00233-x

**Published:** 2020-04-11

**Authors:** Stephanie Jarvis, Kristin Salottolo, Richard Meinig, Chad Corrigan, Nimesh Patel, Matthew Carrick, Mark Lieser, Cassandra Reynolds, David Bar-Or

**Affiliations:** 1 ION Research, 383 Corona St. #319, Denver, CO 80218 USA; 2 Penrose Hospital, 2222 North Nevada Ave, Colorado Springs, CO 80907 USA; 3 Wesley Medical Center, 550 N Hillside St, Wichita, KS 67214 USA; 4 St. Anthony Hospital, 11600 West 2nd Place, Lakewood, CO 80228 USA; 5 Medical City Plano, 3901 West 15th Street, Plano, TX 75075 USA; 6 Research Medical Center, 2316 East Meyer Blvd, Kansas City, MO 64132 USA; 7 Swedish Medical Center, 501 E Hampden Ave, Englewood, CO 80113 USA

**Keywords:** Pelvic fracture management, Circumferential compression device, Trauma

## Abstract

**Introduction:**

There is a lack of data on the use and effectiveness of pre-hospital pelvic circumferential compression devices (PCCD) as a temporary intervention for pelvic fracture management; they are thought to decrease pelvic volume and hemorrhage but are not without risks. The purpose of this study is to examine pre-hospital PCCD practices at US Level I trauma centers.

**Methods:**

This was a prospective cross-sectional survey of trauma medical directors at US Level I trauma centers. The aim of this study was to describe patterns of pre-hospital PCCD utilization for pelvic fractures. Responses were compared by region, length in time the center was designated Level I, trauma patient volume, pelvic management guideline followed and blood product guidelines. Data were compared using Fisher’s exact and chi-squared tests.

**Results:**

Of the 158 Level I trauma centers invited, 25% responded. All Level I trauma centers use in-hospital PCCDs, whereas 71% of participant’s paramedic agencies trained on pre-hospital PCCD application. Of those, 44% trained to apply pre-hospital PCCDs to all suspected pelvic fractures. A higher proportion of high-volume centers (77%) than low-volume centers (25%) trained on pre-hospital PCCD placement, *p* = 0.06. PCCD practices were not dependent on the trauma center’s region, trauma volume, length in time as a Level I trauma center, or pelvic fracture guideline followed.

**Conclusions:**

There is widespread application of in-hospital and pre-hospital PCCD at US Level I trauma centers, however pre-hospital PCCDs are not applied to all suspected pelvic fractures. Future studies should focus on efficacy, safety, and contraindications for pre-hospital PCCDs.

## Introduction

The use of pelvic circumferential compression devices (PCCD) such as binders, sheets, or wraps, for pelvic fracture management are common. Their use is recommended by the Eastern Association for the Surgery of Trauma (EAST), Western Trauma Association (WTA), Advanced Trauma Life Support (ATLS), the World Society of Emergency Surgery (WSES), and Trauma Quality Improvement Project (TQIP) [1–5]. However, most studies consist of case reports or studies of human cadaveric specimens, with no Level I or II evidence on the effectiveness of PCCDs for pelvic fractures [6]. PCCDs are thought to decrease pelvic volume and limit hemorrhage but they are not without risk for skin damage, internal organ damage, increase in pelvic inlet area, internal rotation, ulceration, and additional fracturing for lateral compression fractures [1, 3, 4, 6–9]. It remains unresolved whether PCCD placement for certain fracture types is contraindicated [6].

Moreover, there is little data available on the use, effectiveness, and safety of PCCDs applied in a pre-hospital setting [6]. The only guideline that states that PCCD can be placed pre-hospital is the WTA guideline, but the WTA guideline also states that PCCDs are contraindicated for lateral compression fractures, which would likely be unknown during pre-hospital application [2, 8]. Given the lack of data on the use and effectiveness of pre-hospital PCCDs, it is important to know current practices at Level I trauma centers. This study aimed to describe PCCD practices and to explore the relationship between Level I trauma center characteristics and practices via National survey.

## Methods

This anonymous cross-sectional survey conducted via SurveyMonkey Inc. (San Mateo, California; www.surveymonkey.com) was approved by the Western Institutional Review Board. Level I trauma centers were identified from the American College of Surgeons (ACS) website and the trauma medical director was identified via telephone or the center’s website. Six email invitations were sent to 158 trauma medical directors at all ACS-verified Level I trauma centers, to view invitees, see Appendix 1. SurveyMonkey kept responses anonymous while tracking participation so that only those who did not participate, or those who did not reject the invitation to participate, were sent reminder email invitations. Responses to survey questions were not linked to those who participated. The trauma medical director was called before sending the final two invitations to confirm they received the invitation.

Forty-six questions were asked; questions pertaining to this manuscript can be found in Table 1 in Appendix 2. SurveyMonkey’s ‘skip logic’ skipped irrelevant questions; for example, if the paramedic agency did not train on pre-hospital PCCD placement, then the participant was not asked if the paramedic agency trained to apply pre-hospital PCCDs to all pelvic fractures. Participants were also able to skip questions for any reason; therefore, the denominator reported for each question varies based on the number of participants who responded. Level I trauma center characteristics have been previously reported and included the US census bureau region, volume of trauma admissions in 2017, and length in time the center has been an ACS-verified Level I trauma center [10, 11]. The volume of trauma admissions was dichotomized as high-volume (≥1501 admissions) and low-volume (≤1500 admissions).

Responses to survey questions were summarized as proportions (counts). The relationship between survey responses on the application of pre-hospital PCCDs with the Level I trauma center’s characteristics and guidelines were compared using Fisher’s exact or chi-squared tests when appropriate, alpha = 0.05. Level I trauma center’s characteristics included: the region of the participating trauma center, the volume of trauma admissions in 2017, and the length of time in years that the participating center has been an ACS-verified Level I trauma center. Guideline characteristics of interest included: the guideline followed, the year that the guideline followed was published, and the inclusion of blood products in the guideline followed.

## Results

Of the 158 invited to participate in the survey, 25% (40/158) responded and 90% (36/40) completed the survey. Seventy-one percent (25/35) of participants reported that their paramedic agency required training on PCCD application (Table 1 in Appendix 2). Of those, 44% (11/25) of paramedic agencies trained to apply pre-hospital PCCDs to all suspected pelvic fractures. Although not everyone used pre-hospital PCCD, all participants [100% (27/27)] utilized in-hospital PCCD. The priority treatment sequence for hemodynamically unstable pelvic fractures was previously reported; almost all of the participants, [89% (24/27)], applied PCCD first [10]. There were two participants who applied PCCD following angioembolization and external fixation and one participant who applied PCCD following angioembolization, external fixation, and exploratory laparotomy.

Most participants [43% (9/21)] followed the EAST guideline for pelvic fracture management (Table 1 in Appendix 2). Guidelines followed were published between from 1995 to 2018, although most followed a guideline published in 2016 [21% (3/14)]. A majority of Level I trauma centers [78% (28/36)] followed a guideline that included the administration of blood products; 69% (25/36) using the massive transfusion protocol (MTP). Thirty-three percent (12/36) of Level I trauma centers guidelines included other blood products or fluids, outside of the MTP in their pelvic fracture management guideline.

All participants following TQIP and ATLS had paramedic agencies who required training on pre-hospital PCCD placement; whereas 67% (4/6) of those following the WTA guideline had paramedic agencies who required training on pre-hospital PCCD placement (Table 2 in Appendix 2). There was a higher proportion of high-volume centers [77% (24/31)] than low-volume centers [25% (1/4)] that had paramedic agencies who trained on PCCD placement; however, this was not statistically significant, *p* = 0.06. Paramedic agency training on PCCD placement was not dependent on the length of time the trauma center was an ACS-verified Level I trauma center, *p* = 0.71 or the region, *p* = 0.73. Both the Level I trauma centers’ characteristics and the guideline characteristics did not significantly affect the paramedic agency on the training of PCCDs application to *all* patients with a suspected pelvic fracture (Table 3 in Appendix 2).

## Discussion

The results of this survey show that all participating Level I trauma centers are using in-hospital PCCDs for pelvic fracture management, the majority of trauma center’s paramedic agencies have required training on pre-hospital PCCD placement, but less than half taught to apply pre-hospital PCCDs to all suspected pelvic fractures. PCCD practices were not dependent on the guideline followed, the trauma center’s region, volume of trauma admissions, or length of time as an ACS-verified Level I trauma center.

Although data on the effectiveness of pre-hospital PCCDs is limited, the majority of Level I trauma centers in this study indicated that their paramedic agency required training on pre-hospital PCCD application [1, 9, 12]. The limited data on pre-hospital PCCD application is reflected in the lack of guideline recommendations on pre-hospital placement [1–5]. The WTA guideline is the only guideline that recommends pre-hospital application, however only 67% of those who reported following the WTA guideline also reported their paramedic agency taught paramedics on pre-hospital PCCD application [2]. These results came as a surprise as the hospitals following the WTA guideline had the lowest rates of use of pre-hospital PCCDs.

The WSES guideline states that commercial pelvic binders are more effective in controlling hemorrhage than sheets or wraps, which are frequently used in pre-hospital settings where resources are low and sheets are available [4, 13, 14]. This could be in part due to the ease of application for commercial binders compared to pelvic wraps and sheets [8, 15]. In fact, one study found that commercial binders were secured correctly 100% of the time and typically in under a minute [15]. Alternatively, another study found a low adherence (50%) to PCCD guidelines for both sheets and commercial devices, but did not report the adherence rate among PCCDs applied in a pre-hospital setting [8, 13]. A common application error is that PCCDs are not placed over the great trochanter, however sheets are sometimes wrapped too loose, too tight, or tied in a knot, when it is recommended to secure PCCDs using clamps [16].

Although it has been suggested not to use PCCD on lateral compression fractures, inclusion of fracture type in treatment guidelines could create confusion as first providers have limited diagnostic tools [8]. One case report described hemodynamic instability and extremity shortening after pre-hospital PCCD placement and hypothesized additional fracturing was the cause [7]. Interestingly this case presented with an absence of a lateral compression fracture, a fracture type thought to be worsened by PCCDs [2, 8]. Another study found 11% of cases experienced increased displacement or deformities after PCCD placement [8]. Even though early PCCD application is thought to decrease the number of transfusions required, this may only occur for specific fracture types, which may not be known in the pre-hospital setting [1, 4, 13, 17]. Matched data comparing blood transfusions for patients who had a pre-hospital PCCD placed and who did not have a pre-hospital PCCD placed is needed to determine if pre-hospital PCCD placement is associated with a risk for blood transfusions.

### Limitations

The response rate of 25% (40/158) was a limitation. Some may have responded based on memory despite survey instructions to have their guideline available. We did not collect data on what qualifies a patient for pre-hospital PCCD placement at paramedic divisions that do not train to apply pre-hospital PCCDs to all suspected pelvic fractures. Additionally, we did not ask if the individual trauma center has a paramedic division specific to their hospital or are at all involved with training of paramedic staff in any capacity. Therefore, some of the participants who responded that their paramedic agency does not train on PCCD application may not have a paramedic agency or may not have knowledge of the paramedic training practices.

## Conclusions

There was no specific guideline followed by all US Level I trauma centers responding to this survey. PCCD practices did not vary based on pelvic fracture guideline followed, region, volume of trauma admissions, or length of time as an ACS-verified Level I trauma center. There is widespread use of pre-hospital PCCD application at US Level I trauma centers and all participating centers utilized in-hospital PCCDs, primarily as the first management approach for hemodynamically unstable pelvic fractures. However, the results of this survey show that pre-hospital PCCDs are not uniformly applied to all suspected pelvic fractures and that a majority of hospitals utilizing pre-hospital PCCDs are selecting specific patients for placement.

## Data Availability

Data for this study is stored on Sharefile, an electronic HIPAA and HITECH-compliant platform that ensures all transmissions are fully encrypted, end-to-end. The datasets used for analysis for the current study are available from the corresponding author on reasonable request.

## Appendix 1

### Level I Trauma Centers Invited to Participate in the Survey

Albany Medical Center, Banner University Medical Center – Tucson, Banner University Medical Center Phoenix, Barnes-Jewish Hospital, Baylor University Medical Center at Dallas, Baystate Medical Center, Beaumont Hospital - Royal Oak Campus, Bellevue Hospital Center, Ben Taub Hospital - Harris Health System, Beth Israel Deaconess Medical Center, Boston Medical Center, Brigham and Women’s Hospital, Bronson Methodist Hospital, Brooke Army Medical Center, Carilion Roanoke Memorial Hospital, Carolinas Medical Center, Cedars-Sinai Medical Center, Charleston Area Medical Center, Christiana Care Health System, Cleveland Clinic Akron General, Community Regional Medical Center, Cooper University Health Care, Dartmouth-Hitchcock Medical Center, Dell Seton Medical Center at the University of Texas, Denver Health Medical Center, Detroit Receiving Hospital, Dignity Health Chandler Regional Medical Center, Dignity Health St. Joseph’s Hospital and Medical Center, Duke University Hospital, East Texas Medical Center Tyler, Erie County Medical Center, Eskenazi Health, Froedtert Hospital, George Washington University Hospital, Grady Memorial Hospital, Grant Medical Center, Greenville Memorial Hospital, Harbor UCLA Medical Center, Hartford Hospital, Hennepin County Medical Center, Henry Ford Hospital, Highland Hospital/A member of Alameda Health System, HonorHealth John C. Lincoln Medical Center, HonorHealth Scottsdale Osborn Medical Center, Howard University Hospital, Hurley Medical Center, Indiana University Health Methodist Hospital, Inova Fairfax Hospital, Intermountain Medical Center, Iowa Methodist Medical Center, Jackson Memorial Hospital, Jacobi Medical Center, Jamaica Hospital Medical Center, JPS Health Network, Kendall Regional Medical Center, LAC + USC Medical Center, Legacy Emanuel Medical Center, Lincoln Medical and Mental Health Center, Loyola University Medical Center, Maine Medical Center, Maricopa Integrated Health System - Maricopa Medical Center, Massachusetts General Hospital, Mayo Clinic Rochester Trauma Centers, Medical Center Navient Health, Medical University of South Carolina, MedStar Washington Hospital Center, Memorial Hermann Hospital System – Houston, Memorial Regional Hospital, Mercy Health - St. Elizabeth Youngstown Hospital, Mercy Health - St. Vincent Medical Center, Methodist Dallas Medical Center, MetroHealth Medical Center, Miami Valley Hospital, Morristown Medical Center, Nassau University Medical Center, Nebraska Medicine - Nebraska Medical Center, New Jersey Trauma Center at the University Hospital, New York Presbyterian Hospital - Weill Cornell Medical Center, New York-Presbyterian – Queens, North Memorial Health Hospital, Northwell Health North Shore University Hospital, Northwell Health Staten Island University Hospital, NYC Health and Hospitals – Elmhurst, NYC Health and Hospitals - Kings County, NYU Langone Hospital – Brooklyn, NYU Winthrop Hospital, Oregon Health & Science University, OU Medical Center, Palmetto Health Richland, Parkland Health & Hospital System, Penrose Hospital, ProMedica Toledo Hospital, Regions Hospital, Rhode Island Hospital, Richmond University Medical Center, Robert Wood Johnson University Hospital, Ronald Reagan UCLA Medical Center, Santa Barbara Cottage Hospital, Santa Clara Valley Medical Center, Scott & White Memorial Hospital – Temple, Scripps Mercy Hospital, Sparrow Hospital, Spectrum Health - Butterworth Hospital, SSM Health Saint Louis University Hospital, St. Anthony Hospital, St. Joseph Mercy Hospital - Ann Arbor, St. Vincent Indianapolis Hospital, Stanford Health Care, Stony Brook Medicine, Summa Akron City Hospital, Swedish Medical Center, Tampa General Hospital, Medical City Plano, The Ohio State University Wexner Medical Center, The Queen’s Medical Center, The University of Kansas Hospital, The University of Toledo Medical Center, Tufts Medical Center, UC Irvine Health, UC San Diego Medical Center, UMASS Memorial Medical Center, University Health System - San Antonio, University Health-Shreveport, University Hospitals Cleveland Medical Center, University Medical Center – Lubbock, University Medical Center New Orleans, University Medical Center of El Paso, University Medical Center of El Paso, University Medical Center of Southern Nevada, University Medical Center of Southern Nevada, University of Alabama at Birmingham Hospital, University of Arkansas for Medical Sciences, University of California, Davis Medical Center, University of Cincinnati Medical Center, University of Iowa Hospitals & Clinics, University of Kentucky Albert B. Chandler Hospital, University of Louisville Hospital, University of Michigan Health System, University of Missouri Health System, University of New Mexico Hospital, University of North Carolina Hospital, University of Rochester Medical Center/Strong Memorial Hospital, University of Tennessee Medical Center, University of Texas Medical Branch, University of Utah Health Care, University of Vermont Medical Center, University of Virginia Health System, University of Wisconsin Hospital and Clinics Authority, Upstate University Hospital, Vanderbilt University Medical Center, Via Christi Hospitals – Wichita, Vidant Medical Center, Virginia Commonwealth University Medical Center, Wake Forest Baptist Medical Center, WakeMed Health & Hospitals, Wesley Medical Center, West Virginia University Hospitals-J.W. Ruby Memorial Hospital, Westchester Medical Center, Yale-New Haven Hospital, and Zuckerberg San Francisco General Hospital and Trauma Center.

## Appendix 2

**Table 1:** Survey Questions and Participant Responses

Question	Responses	% (n)	n total
Is your hospital’s guideline for pelvic fracture management based on a published guideline or algorithm?	EAST	43% (9)	21
	WTA	29% (6)	
	TQIP	14% (3)	
	ATLS	10% (2)	
	Other	5% (1)	
In what year was the guideline that your hospital is using for the management of pelvic fractures published?	1995	7% (1)	14
	2008	7% (1)	
	2010	7% (1)	
	2011	7% (1)	
	2013	14% (2)	
	2014	7% (1)	
	2015	14% (2)	
	2016	21% (3)	
	2017	7% (1)	
	2018	7% (1)	
Does your hospital guideline for pelvic fracture management include the transfusion of blood products?	Yes	78% (28)	36
	No	22% (8)	
Does your hospital guideline for pelvic fracture management specify when to consider the massive transfusion protocol?	Yes	69% (25)	36
	No	31% (11)	
Does your hospital’s guideline for pelvic fracture management specify to provide blood products or fluids to the patients, not including massive transfusion protocol products?	Yes	33% (12)	36
	No	67% (22)	
Does your hospital use PCCD for hemodynamically unstable pelvic fractures?	Yes	100% (27)	27
	No	0% (0)	
Does your hospital’s paramedic agency require training for the application of pre-hospital PCCDs? PCCDs include pelvic binders and pelvic sheets.	Yes	71% (25)	35
	No	29% (10)	
Does the paramedic training state to apply pre-hospital PCCDs on all patients with a suspected pelvic fracture?	Yes	44% (11)	25
	No	56% (14)	

*EAST* eastern association for the surgery of trauma, *WTA* western trauma association, *TQIP* trauma quality improvement project, *ATLS* advanced trauma life support, *PCCD* pelvic circumferential compression device

**Table 2:** Pre-hospital PCCD Use for Pelvic Fracture Management at Level 1 Trauma Centers

	Paramedic Training on PCCDs *n* = 25	No Paramedic Training on PCCDs *n* = 10	p
Guideline followed			
EAST	89% (8)	11% (1)	0.27
WTA	67% (4)	33% (2)	
TQIP	100% (2)	0% (0)	
ATLS	100% (2)	0% (0)	
MTP in Guideline?			
Yes	64% (16)	80% (8)	0.47
No	36% (9)	20% (2)	
Other Blood Products in Guideline?			
Yes	56% (14)	0% (0)	0.01
No	44% (11)	100% (10)	
Region			
Midwest	80% (8)	20% (2)	0.73
Northeast	57% (4)	43% (3)	
South	67% (8)	33% (4)	
West	83% (5)	17% (1)	
Length in time as an ACS-Verified Level 1 Center			
< 1 year	100% (2)	0	0.71
≥ 1 year to 2 years	60% (3)	40% (2)	
≥ 2 years to 5 years	86% (6)	14% (1)	
≥ 5 years to 10 years	50% (1)	50% (1)	
≥ 10 years	68% (13)	32% (6)	
Volume of Trauma Admissions in 2017			
Low-volume	25% (1)	75% (3)	0.06
High-volume	77% (24)	23% (7)	

*EAST* eastern association for the surgery of trauma, *WTA* western trauma association, *TQIP* trauma quality improvement project, *ATLS* advanced trauma life support, *PCCD* pelvic circumferential compression device, *MTP* massive transfusion protocol, *ACS* american college of surgeons

**Table 3:** Pre-hospital PCCD Use for All Pelvic Fractures at Level 1 Trauma Centers

	Apply PCCD to All Pelvic Fractures *n* = 11	Do not Apply PCCD to All Pelvic Fractures *n* = 14	p
Guideline followed			
EAST	38% (3)	63% (5)	0.89
WTA	50% (2)	50% (2)	
TQIP	0% (0)	100% (2)	
ATLS	50% (1)	50% (1)	
MTP in Guideline?			
Yes	55% (6)	71% (10)	0.43
No	45% (5)	29% (4)	
Other Blood Products in Guideline?			
Yes	45% (5)	43% (6)	> 0.99
No	55% (6)	57% (8)	
Region			
Midwest	63% (3)	38% (5)	0.36
Northeast	75% (3)	25% (1)	
South	25% (2)	75% (6)	
West	60% (3)	40% (2)	
Length in time as an ACS-Verified Level 1 Center			
< 1 year	50% (1)	50% (1)	0.36
≥ 1 year to 2 years	0	100% (3)	
≥ 2 years to 5 years	33% (2)	67% (4)	
≥ 5 years to 10 years	100% (1)	0	
≥ 10 years	54% (7)	46% (6)	
Volume of Trauma Admissions in 2017			
Low-volume	100% (1)	0	> 0.99
High-volume	46% (11)	54% (13)	

*EAST* eastern association for the surgery of trauma, *WTA* western trauma association, *TQIP* trauma quality improvement project, *ATLS* advanced trauma life support, *PCCD* pelvic circumferential compression device, *MTP* massive transfusion protocol, *ACS* american college of surgeons

## Abbreviations

ACS: American College of Surgeons
ATLS: Advanced Trauma Life Support
EAST: Eastern Association for the Surgery of Trauma
MTP: Massive Transfusion Protocol
PCCD: Pelvic circumferential compression device
TQIP: Trauma Quality Improvement Project
WSES: World Society of Emergency Surgeons
WTA: Western Trauma Association
